# Streptococcus agalactiae clinical isolates in Northwest Iran: antibiotic susceptibility, molecular typing, and biofilm formation

**DOI:** 10.3205/dgkh000358

**Published:** 2020-09-29

**Authors:** Mohammad Alipour Shadbad, Hossein Samadi Kafil, Mohammad Ahangarzadeh Rezaee, Marjan Rahnamaye Farzami, Alireza Dolatyar Dehkharghani, Javid Sadeghi, Pourya Gholizadeh, Farzaneh Khodaei, Mohammad Aghazadeh

**Affiliations:** 1 Immunology Research Center, Tabriz University of Medical Sciences, Tabriz, Iran; 2 Student Research Committee, Tabriz University of Medical Sciences, Tabriz, Iran; 3 Drug Applied Research Center, Tabriz University of Medical Sciences, Tabriz, Iran; 4 Reference Health Laboratory, Ministry of Health & Medical Education, Tehran, Iran; 5 Biotechnology Research Center, Tabriz University of Medical Sciences, Tabriz, Iran

**Keywords:** biofilm, genotype, drug resistance, pathogenesis, pregnant woman, Streptococcus agalactiae

## Abstract

**Background:** Group B Streptococcus (*S. agalactiae*) is one of the colonizing bacteria in pregnant women which can be a causative agent of meningitis and neonatal sepsis. This organism has also been increasingly related to invasive infections in non-pregnant adults.

**Objective:** In present study, we aimed to characterize the clonality of biofilm-producing *S. agalactiae* isolates from various sources from two different clinical laboratories in Tehran, Iran.

**Materials and Methods:** *S. agalactiae* isolates were collected from community-acquired (CA) and hospital-acquired (HA) infections in pregnant and non-pregnant adults. The antimicrobial susceptibility patterns and biofilm formation ability were determined. In addition, pulse field gel electrophoresis (PFGE) was used to verify the clonal diversity of isolates.

**Results:** Out of the 87 isolates, 15 (16.6%) formed biofilm. The antibiotic resistance rate was 98.85% for clindamycin, 98.85% for tetracycline, followed by 29.88% for erythromycin, 9.19% for moxifloxacin and 6.89% for levofloxacin. The PFGE patterns revealed a total of 16 different clusters consisting of 6 single types (STs).

**Conclusion:** This study evaluated the biofilm formation of clinical *S. agalactiae*, which may be a step towards understanding its role in pathological processes. Biofilm formation was significant only in the hypervirulent ST-17 clone. Intraclonal spread of isolates indicates that a local lineage of isolates is responsible for infection by these bacteria.

## Zusammenfassung

**Hintergrund:** Streptokokken der Gruppe B (*S. agalactiae*) können bei schwangeren Frauen kolonisieren und Meningitis und Sepsis bei Neugeborenen verursachen. Der Erreger wird zunehmend auch mit invasiven Infektionen bei nicht schwangeren Erwachsenen in Verbindung gebracht.

**Zielsetzung:** In der vorliegenden Studie sollte die Klonalität Biofilm-produzierender Isolate von *S. agalactiae* charakterisiert werden, die aus verschiedenen Quellen in zwei klinischen Laboratorien in Teheran, Iran, isoliert wurden.

**Material und Methode:** Die Isolate von *S. agalactiae* wurden bei schwangeren und nicht schwangeren Frauen aus ambulant (CA) und stationär erworbenen (HA) Infektionen gewonnen. Bestimmt wurden die Resistenz und die Biofilmbildung. Die klonale Diversität der Isolate wurde mittels Pulsfeld-Gel-Elektrophorese (PFGE)-Technik bestätigt.

**Ergebnisse:** Von 87 Isolaten bildeten 15 (16,6%) einen Biofilm. Die Resistenzrate betrug für Clindamycin 98,9%, für Tetracyclin 98,9%, für Erythromycin 29,9%, für Moxifloxacin 9,2% und für Levofloxacin 6,9%. Die PFGE-Muster zeigten 16 verschiedene Cluster, die aus 6 Einzeltypen (STs) bestanden.

**Schlussfolgerung:** Diese Studie untersuchte die Biofilmbildung von klinischen *S. agalactiae* Isolaten, die ein Schritt zum Verständnis in der Pathogenese sein könnte. Die Biofilmbildung war nur bei einem hypervirulenten ST-17-Klon signifikant. Die intraklonale Ausbreitung der Isolate deutet darauf hin, dass eine lokale Linie von Isolaten für die Infektion durch diese Bakterien verantwortlich ist.

## Introduction

*Streptococcus agalactiae* (Group B Streptococci; GBS) can be a common colonizer of the urogenital and gastro-intestinal tracts of up to 40% of healthy individuals [1]. In addition, GBS is able to become a life-threatening pathogen, which can cause invasive infections among human neonates of healthy women in whom it is part of their normal flora. Moreover, GBS is a main cause of mortality and morbidity in the elderly and immuno-compromised adults [2], [3].

Some virulence factors are critical for GBS biofilm formation and colonization, which is the first step in pathogenesis. Proteins which facilitate binding to host-cell surface elements include laminin-binding protein (Lmb), serine-repeat rich proteins (Srr-1 and Srr-2), fibrinogen-binding proteins (FbsA, FbsB, and ScpB), and pili [4]. Likewise, the antigenic difference and chemical composition of the polysaccharide capsule has been associated with virulence, while biofilm formation and survivability in various environments were also found to be critical, principally given colonization. Biofilm is an accumulation of cells in a distinctly sessile state, enclosed in a self-generated matrix composed of polysaccharides as well as protein and extra cellular DNA [5]. Biofilm production could be a main virulence determinant in several bacterial pathogens that have been associated with colonization and infection progression [6], [7]. Biofilms suggest protection in hostile environments containing immune cells, antimicrobials and extreme pH, thus promoting the conservation of a microorganism population which can then cause heavy colonization and chronic infection [8], [9], [10]. In addition, certain environmental conditions within biofilms may promote selective pressure that can increase pathogenicity through the increase of genotypic and phenotypic determinants [8], [11]. Several studies have demonstrated that pili play a main role in biofilm formation in GBS, which is encoded by one or two distinct pilus islands (PI), including PI-1 and PI-2 [1], [12]. Despite recent studies, the effect of PI in biofilm formation and pathogenesis of the bacteria is not known. In this study, we characterized the antibiotic resistance pattern, biofilm formation of GBS isolates, and typing by pulse field gel electrophoresis (PFGE). 

## Methods and materials

### Sample collection

In this study, we used 87 *S. agalactiae* isolates which had been previously collected from community-acquired (CA) and hospital-acquired (HA) infections in pregnant and non-pregnant adults from two different clinical laboratories in Tehran and Karaj between 2014 and 2015. The isolates were identified by routine biochemical phenotypic tests, including hemolytic activity and colony morphology on sheep blood agar medium, direct smear after gram staining, catalase reaction, hippurate hydrolysis, CAMP test, esculin, pyrrolidonyl arylamidase and leucine aminopeptidase activity testing, susceptibility to bacitracin, and reactivity with Lancefield group B-specific antiserum [13]. In addition, the isolates were identified by scp gene, which confirmed GBS at the species level. The isolates were stored in trypticase soy broth containing 15% glycerol at –20°C.

### Antimicrobial agents and MIC determination

Antibacterial susceptibility testing of isolates was performed in our previous study [14] according to the Clinical and Laboratory Standards Institute (CLSI, 2017) guideline [15]. All isolates were evaluated on antibiotic disks including penicillin (10 µg), vancomycin (30 µg), tetracycline (30 µg), erythromycin (15 µg), moxifloxacin (5 µg), levofloxacin (5 µg), and clindamycin (2 µg). In addition, the minimum inhibitory concentration (MIC) of erythromycin and clindamycin was determined for each isolate was according to CLSI 2012 [15]. 

### Biofilm assay

The ability of isolates to adhere and form biofilms on solid surfaces (biofilm production) was determined according to previous studies [6], [16]. The biofilm assay was conducted in 96-well polystyrene flat-bottom microtiter plates. GBS isolates were grown overnight in Todd-Hewitt broth (THB) containing 1% glucose and subsequently diluted 1:20 with fresh medium. 100 µl of cultures were added to each well first rinsed three times with phosphate buffered saline (PBS). Wells filled with growth medium without inoculation were included as negative controls. The plates were incubated without shaking at 37°C with 5% CO_2_ for 18 h. The growth of the isolates was evaluated using spectrophotometry to measure the absorbance of cultures in the wells at 600 nm before biofilm quantification. To determine biofilm formation, each well was washed twice with 200 µl PBS, and then stained with 100 µl crystal violet for 10 min. All remaining crystal violet was rinsed off with PBS three times, and 200 µl of 95% ethanol was used to solubilize bound crystal violet. Biofilm formation was quantified by measuring absorbance of the solution at 540 nm using a plate reader (Tecan, Infinite M200); measurements were calculated as the sample value minus the medium (blank) control. Biofilm was formed for each isolate in triplicate and the absorbance mean was calculated and reported. The median value of all of the isolates tested at 600 nm was >1.0, which indicated that the isolates produced a strong biofilm also between 0.5–1.0 moderate and less than 0.5 poor biofilm.

### Molecular typing by PFGE 

Genomic DNA of all GBS isolates was extracted and prepared in agarose plugs as described previously [17]. The genomic DNA was digested with 30 U of SmaI restriction enzyme (Amersham Biosciences, UK). The DNA fragments were separated by PFGE in 1.2% agarose gels, with pulse times of 3.5 to 45 s for 12 h and 1 to 5 s for 8 h at 14°C and 6 V/cm in a CHEF-DRIII system (Bio-Rad Laboratories, USA). The lambda phage concatemers were run at the same time as a size marker. The gels were stained with ethidium bromide and visualized by a gel documentation system. PFGE patterns were analyzed by the unweighted-pair group method with arithmetic averages (UPGMA) using Bionumerics software (Applied Maths, Sint-Martens-Latem, Belgium) to create dendrograms. The results were interpreted according to the criteria proposed by van Belkum et al. [18]. 

### Ethics statement

This study was approved by the ethics committee of Tabriz University of Medical Science. 

### Statistical analysis

Statistical analysis was done using SPSS Version 19.0. Statistical significance was assessed using repeated-measures ANOVA (OD values). The results were expressed as a means ± standard deviation. Statistical significance was set at P<0.05.

## Results

In all isolates, the *scp* gene identified and confirmed GBS strain. The most isolates were obtained from urine samples of asymptomatic female patients (36.8%) and vaginal secretions of pregnant women (28.7%), followed by blood (14.9%), urinary tract infections (14.9), and spermatic fluid (4.6%). The mean age of individuals was 36 years (17–80 years). Antibiogram patterns of isolates showed that all isolates were susceptible to penicillin and vancomycin. Most isolates were resistant to tetracycline (98.9%), followed by erythromycin (29.9%), clindamycin (18.9%), moxifloxacin (9.2%), and levofloxacin (6.9%). D-zone revealed 2 isolates with inducible clindamycin resistance. 

The results of this study showed that the observed MICs of the 15 biofilm-producing *S. agalactiae* ranged from 0.32 µg/mL for erythromycin, 0.5–16 µg/mL (MIC_50_=0.5 µg/ml, and MIC_90_=2 µg/ml) for clindamycin, 0.125–16 µg/mL (MIC_50_ and MIC_90_=0.5 µg/ml) for erythromycin. Particularly the rates of resistance to erythromycin and clindamycin were not significantly different compared to the other PFGE type or serotypes.

### Biofilm formation

Different biofilm formation abilities were observed in *S. agalactiae* isolates were observed. A baseline calculation of 3 standard deviations revealed no, weak and strong biofilm formers. In total, biofilm formation was observed in only 15 (16.6%) isolates. There was no significant correlation between formation of biofilm, antibiotic resistance, and clone type in the population studied. The erythromycin resistance rate was 70% (17/24) in biofilm-positive isolates, but biofilm formation ability was unrelated to resistance to other antibiotics, such as penicillin and clindamycin. In this study, the relationship between different GBS serotypes and biofilm formation capacity was investigated, the highest number of strong biofilm-forming isolates were found to belong to serotype III; however, not all type III isolates showed good biofilm formation proclivity. Furthermore, the subset of type III strong biofilm-forming strains belonged to the hypervirulent ST-17 clone. 

### Pulsed-Field Gel Electrophoresis Profiling

**Figure 1:**
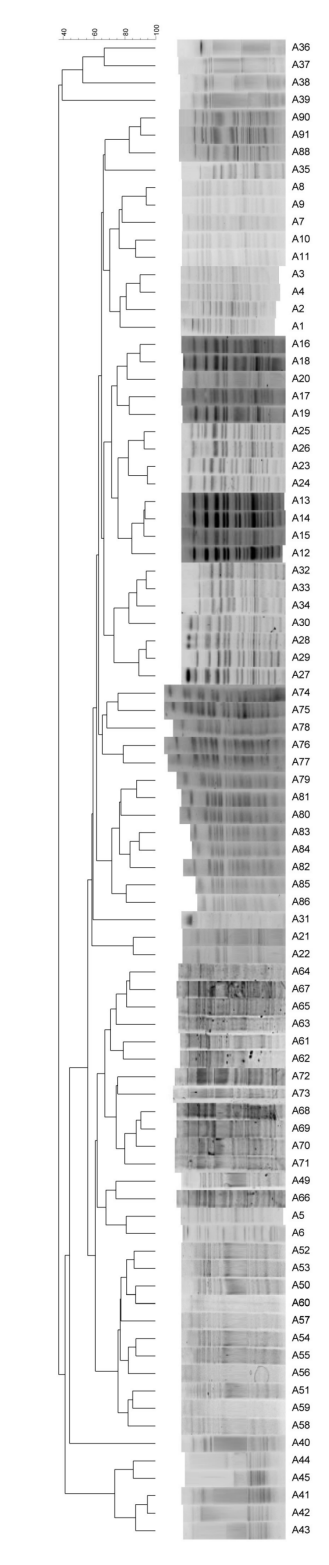
Dendrogram and PFGE patterns of all 87 *S. agalactiae* isolates following digestion with SmaI restriction enzyme

All isolates yielded interpretable PFGE profiles following *SmaI* digestion. PFGE-based clusters were defined as isolates with 75–80% relatedness on a dendrogram created using the Dice coefficient and unweighted pair-group method with arithmetic averages (UPGMA) (Figure 1 (Fig. 1)). The isolates in this study were grouped in 11 PFGE clusters. The remaining isolates (n=6) had unique profiles, presenting less than 80% intraclonal similarity. 

## Discussion

In pathogenic bacteria, stable colonization normally involves the formation of biofilm and pili, which cause advanced bacterial aggregation and attachment to host surfaces. Asymptomatic GBS colonization of maternal vaginal mucosa is a high risk element for meningitis and sepsis in neonates. Because GBS is covered with pili, it was of interest to examine the possible role of pili in the colonization and biofilm formation in GBS strains [19]. In our previous study, we found that three pilus types (1, 2a, and 2b) in GBS isolates are encoded by three distinct genetic islands and at least one island was found in all isolates [14]. We also found a relationship between biofilm production and pilus PI2a, as explained in other studies [1], [19]. We also observed in this study that type 2a pili play a more significant role in biofilm formation in GBS than do types 1 and 2b [2]. 

Quantitative biofilm investigation disclosed unstable biofilm forming ability in GBS isolates from some sources, where a large proportion (56%) of GBS isolates collected from asymptomatic carriers produced biofilm, compared to those from symptomatic patients (24.3%). Similar to our study, Kaur et al. [3] reported that the variation in biofilm-forming ability between GBS isolates from asymptomatic pregnant women was statistically significant (P<0.05). 

In a previous study, we detected the various serotypes of all isolates by Multiplex PCR assay [14]. In addition, highly significant variation in biofilm formation ability among different GBS serotypes was found, which was similar to the findings of Lembke et al. [20], [21]. A majority of GBS isolates with strong biofilm formation belong to serotype III ST-17, which can develop ST-biofilm [22].

In this study, no relationship was found between the biofilm formation ability and resistance to other antibiotics, such as erythromycin and clindamycin. These results were consistence with the results of a study by Jiang et al. [23]. 

We examined the relevance of this tool for the genotyping of GBS, by testing this method using PFGE previously characterized by six VNTR loci in 90 isolates and serotyping [14], [24]. The results of MLVA and PFGE typing were very similar, but the ability of MLVA for typing was significantly higher than for PFGE [24]. Also, isolates from PFGE types were consistently found to belong to the same capsular serotype as observed with MLVA typing [24]. In this study, there was no relationship between biofilm forming phenotype with PFGE types. 

## Conclusion

Based on phenotypic and molecular testing of *S. agalactiae* isolates in Iran, the results of this study should be applicable for controlling the spread of existing antimicrobiallyresistant bacteria and future active surveillance, as well as early detection of a new, emerging type. The intraclonal spread of isolates indicates that a local lineage of isolates is responsible for infection by these bacteria. 

## Notes

### Competing interests

The authors declare that they have no competing interests.

### Acknowledgment

This research was supported by the Immunology Research Center, Tabriz University of Medical by Mohammad Alipour Shadbad with the same title of this manuscript, Sciences, Tabriz, Iran. This study is based on a database from a Ph.D. thesis registered at the Faculty of Medicine, Tabriz University of Medical Science, registered at the Immunology Research Center Tabriz.

### Informed consent 

All patients participating in this study previously filled out informed consent and were informed about all aspects of the study.
